# Dopamine regulates renal osmoregulation during hyposaline stress *via* DRD1 in the spotted scat (*Scatophagus argus*)

**DOI:** 10.1038/srep37535

**Published:** 2016-11-18

**Authors:** Maoliang Su, Xingjiang Mu, Lang Gui, Peipei Zhang, Jianan Zhou, Jie Ma, Junbin Zhang

**Affiliations:** 1 Key Laboratory of Exploration and Utilization of Aquatic Genetic Resources, Ministry of Education, College of Fisheries and Life Science, Shanghai Ocean University, Shanghai, 201306, China

## Abstract

Dopamine is an important regulator of renal natriuresis and is critical for the adaptation of many animals to changing environmental salinity. However, the molecular mechanisms through which dopamine promotes this adaptation remain poorly understood. We studied the effects of dopamine on renal hypo-osmoregulation in the euryhaline fish *Scatophagus argus* (*S. argus*) during abrupt transfer from seawater (SW) to freshwater (FW). Following the transfer, serum dopamine concentration was decreased, and dopamine activated expression of the dopamine receptor 1 (designated *SaDRD1*) in the kidney, triggering the osmoregulatory signaling cascade. SaDRD1 protein is expressed in the renal proximal tubule cells *in vivo*, and is localized to the cell membrane of renal primary cells *in vitro*. Knockdown of *SaDRD1* mRNA by siRNA significantly increased Na^+^/K^+^-ATPase (NKA) activity in cultured renal primary cells *in vitro*, suggesting that expression of *SaDRD1* may oppose the activity of NKA. We demonstrate that exogenous dopamine enhances the response of NKA to hyposaline stress after transferring primary renal cells from isosmotic medium to hypoosmotic medium. Our results indicate that dopamine regulation *via* SaDRD1 ignited the renal dopaminergic system to balance the osmotic pressure through inhibiting NKA activity, providing a new perspective on the hyposaline adaptation of fish.

Salinity is an important environmental factor that greatly affects the physiology of marine fish, influencing their survival and distribution^1^,^2^. Some teleosts such as *Anguilla anguilla*, *Sarotherodon melanotheron* and *Oreochromis niloticus*, have a limited capability to endure salinity fluctuation after long-term acclimation^3^,^4^,^5^, and are thus restricted in their ability to move between environments. Other species are more tolerant of changes in salinity, such as *Scatophagus argus* (commonly known as the spotted scat). *S. argus* is an estuarine inhabitant notable for its tolerance of salinity fluctuation^6^. It is widely distributed in brackish and marine habitats throughout the Indo-Pacific region^6^, and provides an excellent opportunity for the study of osmoregulation in fish. Remarkably, *S. argus* can survive after being directly transferred from the seawater (SW) to freshwater (FW) and vice versa. Thus, its highly effective osmoregulatory mechanisms deserve further attention^7^.

The chemical messenger dopamine is primarily synthesized in the brain and kidney^8^. It is best known for its roles in the brain, where it is important in the control of locomotion, cognition, and affect neuroendocrine secretion^9^,^10^. The kidney possesses an intrarenal dopaminergic system that is distinct from any neural dopaminergic input. Dopamine concentrations in the serum are normally in the picomolar (pM) range, while renal dopamine levels can reach nanomolar (nM) concentrations^11^. In vertebrates, endogenous renal dopamine plays a critical role in the regulation of sodium, potassium and other electrolytes in the kidney^12^,^13^,^14^. In humans, dopamine dysregulation has been implicated in the pathogenesis of salt-induced elevated blood pressure in some subjects^15^.

Dopamine activity is mediated by its receptors, DRD1–5, which belong to the G protein-coupled receptor (GPCR) family of seven-transmembrane domain receptors^16^,^17^. Based on the molecular structure and receptor pharmacology, dopamine receptors are divided into two classes, D_1_-like (subtypes D1 and D5) and D_2_-like (subtypes D2, D3, and D4) receptors^16^,^18^. Only the D_1_-like receptors, DRD1 and DRD5, are known to play a role in dopamine-regulated maintenance of sodium homeostasis and blood pressure in the kidney^19^.

The lack of specific agonists and antagonists for each D_1_-like receptor subtype has made it challenging to distinguish the individual roles of DRD1 and DRD5 in the kidney, although some progress has been made through other methods^20^,^21^. In rats, blocking or down-regulating the DRD1 activity attenuates the natriuretic response to salt infusion in the kidney, suggesting a role distinct from that of DRD5^22^. DRD1 appears to play a greater role than DRD5 in the D_1_-like receptor-mediated regulation of the cAMP-dependent protein kinase signaling pathway in renal proximal tubule cells, as DRD1 activation increases cAMP production to a much greater extent than DRD5 activation^23^. Furthermore, it is likely that the natriuretic effect of dopamine is due mainly to the DRD1 in nephron segments of human^15^. However, other researches imply that DRD5 may be involved in the maintenance of normal sodium and water balance when dietary salt intake is increased^14^. Furthermore, DRD1 and DRD5 may be able to form a heteromeric receptor, cooperatively regulating sodium transport in renal cells^24^. Together, these studies demonstrate a need for further research to clarify the roles of each D_1_-like receptor subtype in renal osmotic regulation^15^.

Euryhaline fishes offer a valuable model for the study of osmoregulation in the kidney, as they frequently move between areas of high and low salinity, and must rapidly adapt to these conditions. Thus, these fish present a unique opportunity to study physiological responses in osmoregulatory organs as they occur. Most previous studies on euryhaline fish have focused on changes in Na^+^/K^+^-ATPase (NKA) activity during periods of osmotic and ionic stresses^3^,^4^,^25^. Here, we seek to gain a greater understanding of the poorly-understood hormonal control of NKA as this important enzyme responds to changing salinity^26^. In the present study, we studied role of dopamine and the D_1_-like receptors on the renal response of the euryhaline fish *Scatophagus argus* (the spotted scat) to hyposmotic stress. We employed a targeted molecular approach to specifically knock down *SaDRD1* levels in cultured primary *S. argus* kidney cells, and observed the resulting response to osmotic stress in the presence and absence of exogenous dopamine. These results bring us closer to understanding the mechanisms of osmotic regulation in this fish.

## Results

### Dopamine concentration detection *in vivo* and *in vitro*

**Figure 1:**
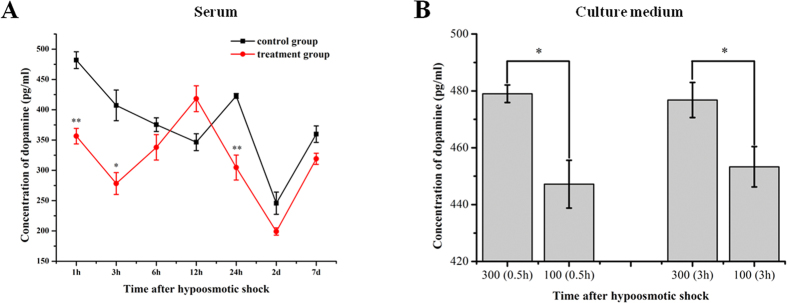
Changes in dopamine concentration following hyposmotic shock *in vivo* and in cultured kidney cells. **(A)** Dopamine concentration in the serum after freshwater shock of *S. argus*. Dopamine contration in the serum of SW-reared *S. argus* was set as the control group, and that of the FW-shock fish was regarded as the treatment group. (**B)** Dopamine concentration in the culture medium of *S. argus* renal primary cells exposed to hypoosmotic conditions (100 mOsmol/L) in *p < 0.05; **p < 0.01.

During acclimation of *S. argus* to FW, dopamine concentrations were significantly reduced in serum from the treatment group (**p < 0.01) at 1 and 3 hours post-transfer (hpt) compared to the control. Serum dopamine in the treated fish then rose, peaking at 12 hpt before declining. Compared with the control group, an increase of about 20% (from 346.5 pg/ml ± 18.2 pg/ml to 418.2 ± 26.7 pg/ml) of dopamine (DA) concentration was observed 12 hpt (*p* = 0.249). Thereafter, dopamine expressions in both groups were similar (Fig. 1).

Dopamine concentration in the culture medium of primary renal cells exposed to hyposmotic shock (100 mOsmol/L) was decreased significantly (*p < 0.05) at 0.5 h and 3 h (Fig. 1).

### Cloning and sequence analysis of *SaDRD1*

**Figure 2:**
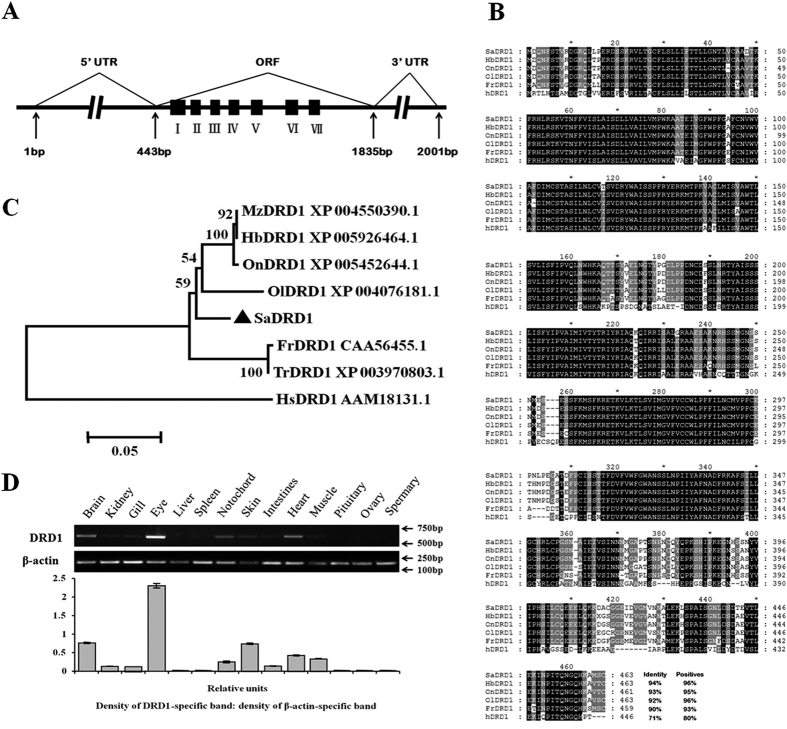
Cloning, sequence analysis and tissue distribution of *S. argus* DRD1 (*SaDRD1*). (**A**) Schematic diagram of the full-length *SaDRD1* cDNA. (**B**) Alignment of the predicted amino acid sequence of SaDRD1 and DRD1 homologs from other species (On: *Oreochromis niloticus*, Tilapia; Hb: *Haplochromis burtoni*, Cichlidae; Ol: *Oryzias latipes*, Medaka; Fr: *Fugu rubripes*, Fugu; Hs: *Homo sapiens*, Human) by Clustal X. (**C**) Phylogenetic analysis of SaDRD1 protein using the neighbor-joining method. Amino acid sequences from different species were obtained from the NCBI GenBank database: *Oreochromis niloticus* (On, Tilapia), *Haplochromis burtoni* (Hb, Cichlidae), *Maylandia zebra* (Mz, Zebra Mbuna), *Oryzias latipes* (Ol, Medaka), *Fugu rubripes* (Fr, Fugu), *Takifugu rubripes* (Tr, Japanese Pufferfish), and *Homo sapiens* (Hs, Human). The numbers at each branch represent the bootstrap values as a percentage of 1000 replicates. Scale bar = 0.05 substitutions per amino acid position. (**D**) Whole tissue distribution of *SaDRD1* mRNA expression in SW-reared *S.argus*, evaluated by sqRT-PCR. *β-actin* was used as an internal control. Densitometric analysis was conducted to determine the *SaDRD1* expression relative to *β-actin*. The results are presented as the mean ± S.E.M. of the data with 3 replicates. N = 15.

A full-length cDNA encoding *S. argus* DRD1, designated *SaDRD1*, was cloned. The full-length of *SaDRD1* cDNA was 2001 bp, containing a 1392-bp ORF, and encoded a precursor protein of 463 amino acids (aa) with 7 transmembrane domains (Fig. 2A). Compared with DRD1 paralog amino acid sequences in other species, SaDRD1 showed 94% identity with *Haplochromis burtoni*, 93% identity with *Oreochromis niloticus*, 92% identity with *Oryzias latipes*, 90% identity with *Fugu rubripes*, and 71% identity with *Homo sapiens* (Fig. 2B). Phylogenetic analysis showed that SaDRD1 was clustered with teleost DRD1 paralogs in a cluster distinct from that of *Homo sapiens* (Fig. 2C).

### Tissue distribution of *DRD1* in *S. argus*

The expression patterns of *SaDRD1* mRNA were examined in SW-reared and FW-shocked *S. argus* using semi-quantitative real-time RT-PCR (sqRT-PCR). In SW-reared fish, *SaDRD1* mRNA was broadly expressed in the eye, brain and heart, and its expression was also observed in osmoregulatory organs, including the kidney and gill (Fig. 2D). Expression patterns of *SaDRD1* mRNA in FW-shocked fish at different time points were shown in Supplementary Fig. S1. In the tissues of central nervous system (brain and notochord), *SaDRD1* expression was detected throughout the period of FW-shock. In osmoregulatory organs (e.g. kidney, gill and skin), expression varied greatly within this period, suggesting that *SaDRD1* was involved in acclimation to FW (Fig. S1).

### mRNA expression of D_1_-like receptors *in vivo* under hyposaline conditions

**Figure 3:**
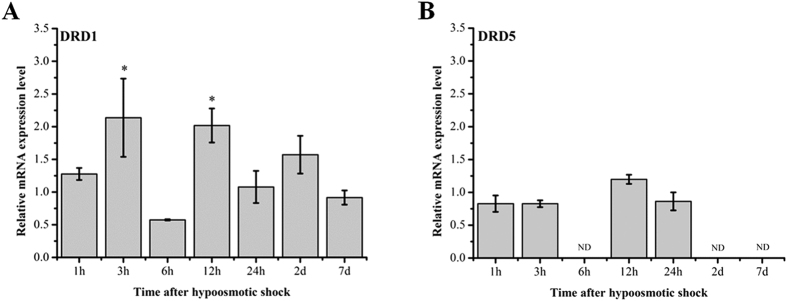
The mRNA expression of *SaDRD1* and *SaDRD5* in kidney during hyposaline shock. The expression levels of *SaDRD1* and *SaDRD5* relative to *β-actin* in kidney during hyposaline shock were evaluated by RT-qPCR, and all the data was normalized to control group (SW → SW). The results are presented as the mean ± S.E.M. of the data with 3 replicates. *p < 0.05; ND means “no detection”.

To determine effects of the hyposaline shock on the expression of D_1_-like receptors in the kidney of *S. argus*, *SaDRD1* and *SaDRD5* mRNA levels were measured by real-time quantitative RT-PCR (RT-qPCR) at different time points after hyposaline shock. *SaDRD1* mRNA expression in the kidney fluctuated highly, with significant upregulation at 3 hpt and 12 hpt (>2-fold) (*p < 0.05) (Fig. 3A). In contrast, *SaDRD5* mRNA in the kidneys of fish transferred from SW to FW exhibited no significant changes, and was undetectable at 6 hpt and 1 dpt (Fig. 3B). The original data (Ct values of *SaDRD1* and *SaDRD5* mRNA expression) was presented in Supplementary Tables S1 and S2.

### Antibody specificity localization of SaDRD1 protein

**Figure 4:**
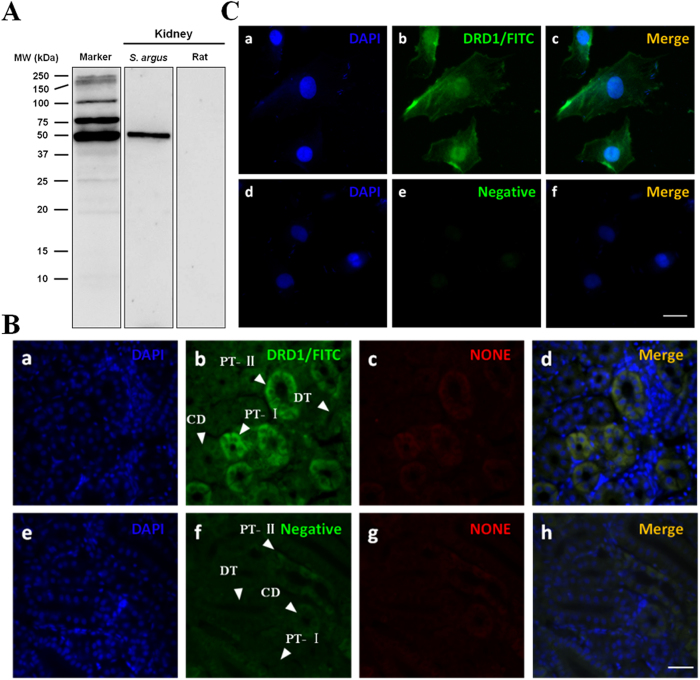
Expression and localiziation of SaDRD1 in SW-reared *S. argus*. (**A**) Western blot showing SaDRD1 (~51 kDa) in the freshly dissected whole kidney of SW-reared *S. argus* was detected to demonstrate its specificity. Immunolocalization of SaDRD1 in the kidney and renal primary cells of *S. argus*. SaDRD1 was visualized using a polyclonal rabbit antibody against SaDRD1 (green), and nuclei were identified by staining with DAPI (blue). (**B**) In tissue sections (a-d), the first segment of the proximal renal tubule (PT-I), second segment of the proximal renal tubule (PT-II), distal renal tubule (DT), and collecting ducts (CD) are indicated. SaDRD1 was localized at PT-I and PT-II. Scale bar = 100 μm. (**C**) In renal primary cells (a-c), SaDRD1 was present at the membranes. Scale bar = 25 μm. In all cases, fluorescence signal was not observed following incubation with pre-immune rabbit serum (B, e-h and C, d-f).

Western blotting was performed to verify the specificity of rabbit anti-SaDRD1 in the kidney of *S. argus* (Fig. 4A). A single band of the expected molecular weight was detected from lysates of *S. argus* kidney, and not observed in rat kidney lysates.

Immunohistochemical experiments were conducted to determine the localization of SaDRD1 protein in the kidney of SW-acclimated *S. argus* and the subcellular localization in renal primary cells under isosmotic culture (approximately 300 mOsmol/L). Renal sections were double-stained with polyclonal rabbit antibody against SaDRD1 (green) and DAPI (blue), while the red channel was set as the background. The arrows and letters in Fig. 4B indicate different tubule segments (PT-I: first segment of the proximal renal tubule; PT-II: second segment of the proximal renal tubule; DT: distal renal tubule; CD: collecting duct). SaDRD1 expression was localized to PT-I and PT-II in fixed kidney sections (Fig. 4B a-d). In cultured kidney cells, SaDRD1 was localized to the cell membrane (Fig. 4C a-c). No fluorescent signal was observed in negative control tissue sections and cell samples (Fig. 4B e-h and Fig. 4C d-f). These results confirm that SaDRD1 is a transmembrane protein.

### Knock-down of *SaDRD1* expression in renal primary cells resulted in increased activity and expression of NKA

**Figure 5:**
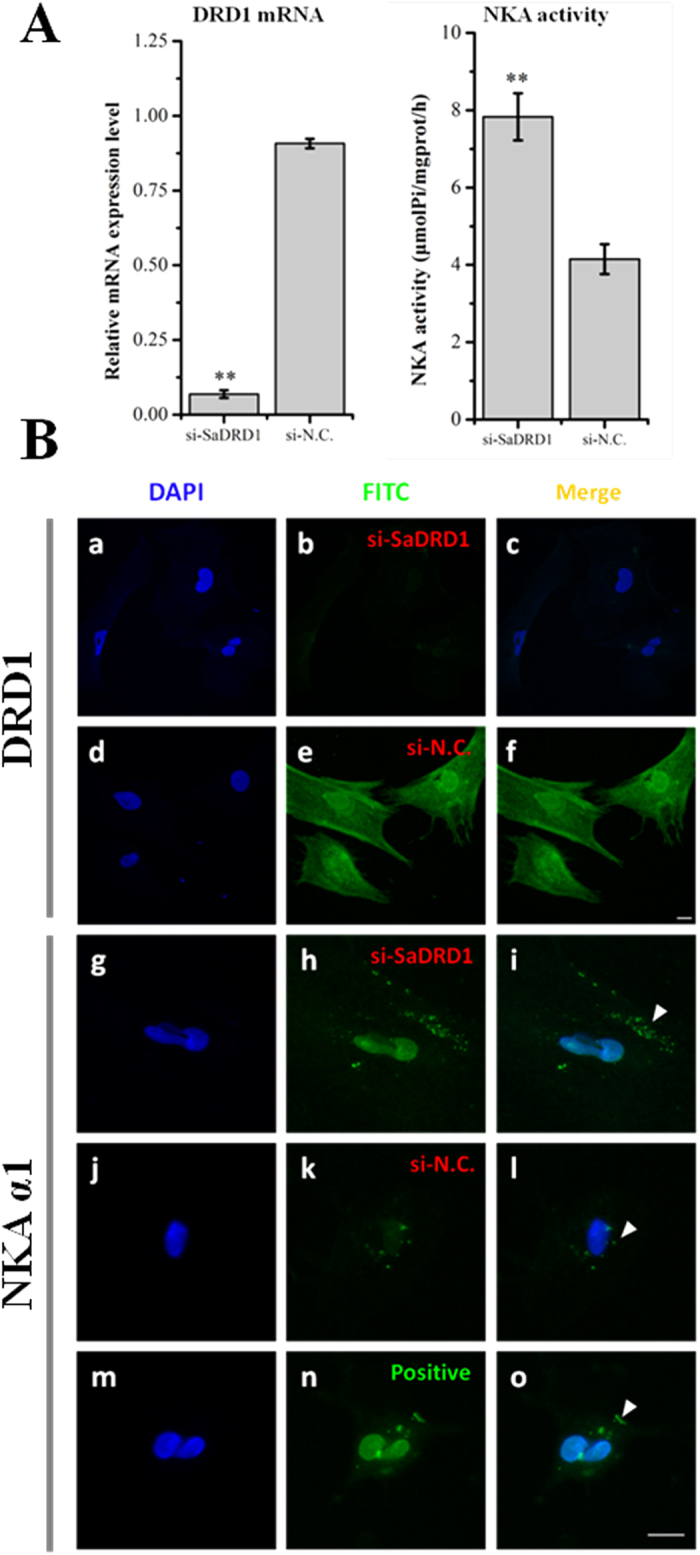
Effect of *SaDRD1* knockdown on Na^+^/K^+^-ATPase (NKA activity) in cultured *S. argus* primary kidney cells (40 hours post-transfection). (**A**) *SaDRD1* mRNA was dramatically reduced by si-*SaDRD1* as determined by RT-qPCR, resulting in the upregulation of NKA activity in cultured primary kidney cells. (**B**) Expression of SaDRD1 protein in renal primary cells was significantly inhibited by si-*SaDRD1* (a-c) compared to the negative control (si-N.C., d-f). Conversely, treatment of renal primary cells with si-*SaDRD1* increased expression of NKA α1 (g-i) relative to control cells (si-N.C., j-l). Positive control without any siRNA treatment was set for locating NKA α1 (m-o). Scale bar = 10 μm.

The relative expression levels of *SaDRD1* were analyzed by RT-qPCR following treatment with si-*SaDRD1*. *SaDRD1* mRNA expression was reduced in renal primary cells by ~13-fold at 40 h post-infection (Fig. 5A, **p < 0.01), and a concomitant increase in NKA activity was observed (7.93 ± 0.61 μmolPi/mgprot/h) compared to the negative control (3.95 ± 0.39 μmolPi/mgprot/h) (Fig. 5A, **p < 0.01). The impact of siRNA on SaDRD1 protein expression was verified by immunofluorescent staining of treated cells. Compared with the negative control (si-N.C., Fig. 5B d-f), the expression of SaDRD1 protein in renal primary cells was significantly inhibited by si-*SaDRD1* (Fig. 5A a-c). si-*SaDRD1* also led to increased NKA in cultured kidney cells at 40 h after RNA interference.

### The change of NKA activity in the kidney of spotted scat under hyposaline conditions

**Figure 6:**
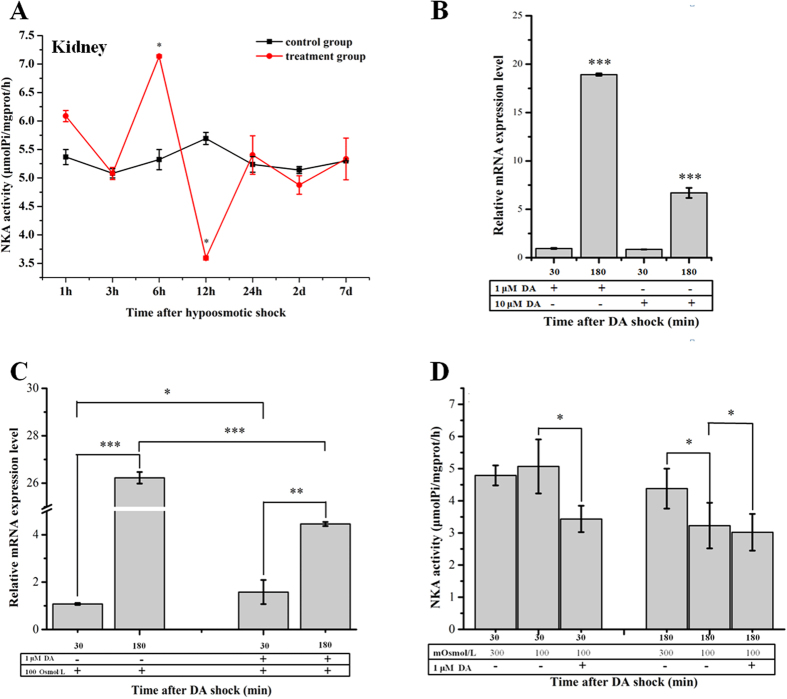
Effect of hyposmotic shock on Na^+^/K^+^-ATPase (NKA) activity in the kidney, and on mRNA expression of *SaDRD1 in vitro*, with and without exogenous dopamine treatment. (**A**) NKA activity in kidneys isolated from fish over a 7 day period following transfer to FW. (**B**) Effects of dopamine (1 μM and 10 μM) on the relative expression of *SaDRD1* mRNA in renal primary cells cultured in isosmotic medium. All the data was normalized to control cells (without dopamine, = 1). (**C**) Changes of *SaDRD1* mRNA expressions for renal primary cells after exposure to the hypoosmotic medium (100 mOsmol/L). (**D**) Effects of hypoosmotic stress and exogenous dopamine on NKA activity in cultured renal primary cells. The hypoosmotic shock and dopamine treatment occurred concurrently. *β-actin* was used as an internal control. The results are presented as the mean ± S.E.M. of the data from 3 replicates (*In vivo*: every biological repeat comprising 5 individuals pooled together; *In vitro*: 3 wells in one experiment). *p < 0.05; **p < 0.01; ***p < 0.001.

Kidneys were isolated from fish that had either been maintained in SW or transferred to FW for varying periods of time. For the first 3 hours after transfer to FW, there was no significant change of NKA activity in the kidney of spotted scat. At 6 hpt NKA activity increased sharply (7.14 ± 0.03 μmolPi/mgprot/h) but transiently, subsequently dropping to 3.62 ± 0.02 μmolPi/mgprot/h at 12 hpt. After 1 day (24hpt), NKA activity returned to levels similar to controls (*p < 0.05) (Fig. 6A).

### Effects of dopamine on expression of *SaDRD1* mRNA and NKA activity *in vitro*

The effects of exogenous dopamine on cultured primary kidney cells were tested under isosomotic and hyposmotic conditions. The addition of exogenous dopamine to cells maintained under the isosmotic condition (300 mOsm/L) caused a significant change of *SaDRD1* mRNA at 180 min after treatment with either 1 μM dopamine (18.9 fold) or 10 μM dopamine (6.7-fold) (***p < 0.001), although no increase was detected at 30 minutes (Fig. 6B).

Exposure of cultured kidney cells to hypoosmotic medium (100 mOsm/L) for 30 minutes did not alter the expression of *SaDRD1* mRNA in the absence of exogenous dopamine. In contrast, when the hypoosmotic shock was accompanied by exogenous dopamine (1 μM), *SaDRD1* mRNA levels increased significantly at this time point (*p < 0.05). After a longer exposure to hypoosmotic medium, for 180 min, *SaDRD1* mRNA expression increased significantly (***p < 0.001) in both the dopamine-treated cells and the control cells. At 180 minutes post-transfer, *SaDRD1* mRNA expression levels were elevated in cells in both the presence and absence of exogenous dopamine. Notably, the increase in expression was greater in the absence of added dopamine at 180 min, showing a 26.3-fold increase, compared to a 4.4-fold increase in the medium containing exogenous dopamine (Fig. 6C). The result indicates that exogenous dopamine initially elevates *SaDRD1* mRNA expression compared to no-dopamine cells when kidney cells are transferred to hyposmotic medium, but that this elevated expression is not sustained.

NKA activity was observed at 30 min and 180 min after exposure to the hyposaline medium in the presence and absence of 1 μM exogenous dopamine. While the NKA activity was not significantly altered in no-dopamine cells at 30 min, treatment with 1 μM exogenous dopamine significantly inhibited NKA activity (from 5.07 ± 0.84 μmolPi/mgprot/h to 3.45 ± 0.71 μmolPi/mgprot/h). The significantly inhibition of NKA activity was observed at 180 min post-transfer in both the dopamine-treated and no-treated cells (Fig. 6D).

## Discussion

Salinity adaptation in euryhaline teleosts is a complex process involving physiological responses in several osmoregulatory organs. The endocrine system mediates many of these processes in order to maintain salt and water balance when fish are challenged with changing environmental salinity^27^,^28^. Dopamine, a catecholamine hormone, directly inhibits Na^+^ transporters, thereby favoring natriuresis through paracrine and autocrine pathways^29^,^30^. Dopamine accumulates in the hypothalamus of tilapia (*Sarotherodon mossambicus*) during SW to FW adaptation^31^. Dopamine is known to inhibit the expression of prolactin, which has been identified as a major osmoregulatory hormone during SW acclimation^32^. However, little is understood about the direct effects of dopamine on the regulation of osmotic balance in the peripheral non-neuronal tissues of teleost fishes^32^.

In mammals, dopamine is synthesized in dopaminergic neurons of the brain, where it serves as a neurotransmitter. A substantial amount of dopamine circulates in the bloodstream, but its origins and functions outside of the brain are not entirely clear^11^,^33^. Due to the presence of blood-brain barrier, dopamine synthesis and function in peripheral areas is expected to be independent of its role in the brain. We have chosen to study the role of dopamine in the transition of fish from high to low salinity conditions, in order to increase our understanding of the role of this neurotransmitter in osmotic regulation in vertebrates. Unlike mammalian models such as rats and mice, for which many cell lines are available, few such resources exist for fish^34^,^35^. Thus, we have developed a protocol for the culture of primary renal cells in our laboratory over the past 3 years^36^, which we have used here to reveal molecular and physiological insights into dopamine function during hypoosmotic acclimation.

In the present study, the serum dopamine level of *S. argus* decreased significantly during the first 24-hours after transfer into FW, with the exception of the 12 hpt collection (Fig. 1). This 12 hpt result parallels findings from one of our previous studies, in which we observed that serum osmotic pressure in *S. argus* returned to normal levels at 12 h after SW to FW shock, following an initial decrease^7^. This phenomenon indicates that during FW acclimation, the serum dopamine in *S. argus* is associated with the short-term regulation of osmotic balance.

Several tissues can secrete dopamine into the circulatory system, and the pancreas is regarded as an important source of non-neuronal dopamine in the rat^37^. Some studies support the view that in humans, free (unconjugated) dopamine in the serum is derived largely from sympathetic noradrenergic nerves^38^. The fish kidney is regarded to play an important role in ionic regulation during environmental adaptation, and during freshwater acclimation, the primary function of the kidney is to drain excess water and retain sodium for the blood to maintain homeostasis^39^. Thus, we were interested in the ability of the fish kidney, and cultured renal cells, to produce and respond to dopamine.

Renal primary cells of *S. argus* were employed here to explore the role of dopamine in osmoregulation *in vitro*. After cells were exposed to hypotonic culture medium (100 mOsmol/L), dopamine contents in the culture medium were reduced substantially at 0.5 h and 3 h in comparison with those exposed to isosmotic culture medium (Fig. 1). This demonstrates the synthesis and release of dopamine can be achieved in the renal cells of *S. argus*. Our results indicate that the renal contribution to serum dopamine may play a prominant role. In the kidney, dopamine can be synthesized and secreted by renal tubular cells and transported to target organs or tissues by hemodynamic mechanisms^22^,^40^.

In the kidney, the natriuretic effect of dopamine is mediated by inhibition of sodium transporters (e.g. NKA) *via* activation of D_1_-like receptors^12^. It is accepted that D_1_-like receptors (DRD1 and DRD5) are the principal mediators of the natriuretic effects of dopamine^19^,^41^. However, roles of DRD1/DRD5 in renal osmotic homeostasis are equivocal^14^. In present study, the mRNA expression of DRD5 in the kidney remained constant during 24 hours after the transfer from SW to FW, and was undetectable at later time points (Fig. 3B). In contrast, FW shock significantly increased renal *SaDRD1* expression (Fig. 3A). Thus, DRD1 is the primary subtype of D_1_-like receptors regulating dopamine-mediated FW adaption in *S. argus*.

The *SaDRD1* gene is predicted to encode a protein of 463 amino acids with 7 transmembrane domains (Fig. 2A). Sequence alignment indicates that SaDRD1 is highly homologous with the DRD1 protein of other vertebrate species (Fig. 2B). The phylogenetic tree in Fig. 2C reveals that SaDRD1 is clearly grouped with DRD1 homologs from other teleosts.

A 127 aa extracellular region of SaDRD1 was chosen for antigen preparation, and the specificity of SaDRD1 antibody was tested by western blot analysis. The anti-SaDRD1 recognized a single band of about 51 kDa in the kidney of *S. argus* (Fig. 4A), the predicted molecular size of DRD1 in the opossum rat^42^. There was no cross-reactivity between anti-SaDRD1 antibody and rat DRD1 (Fig. 4A), which means the high specificity of SaDRD1 antibody. Immunohistochemistry experiment with this antibody showed that SaDRD1 expression is predominantly localized to the renal proximal tubule of *S. argus* (Fig. 4B a-h). Staining of renal primary cells of *S. argus* showed that SaDRD1 is localized to the cell membrane (Fig. 4B i-n), a finding similar to earlier reports in rats^43^.

We assessed changes of *SaDRD1* expression in *S. argus* during hypo-osmostic acclimation *in vitro* and *in vivo*, respectively. *In vivo*, renal *SaDRD1* mRNA expression was significantly up-regulated at 3 hpt and 12 hpt, and was relatively stable after 12 hpt (Fig. 3A). In renal primary cells, *SaDRD1* mRNA expression was increased more than 25-fold at 3 h following transfer from isotonic medium to hypoosmotic medium (Fig. 6C). The expression pattern of *SaDRD1* in response to hypoosmotic stress was opposite to renal NKA activity both *in vivo* and *in vitro* (Fig. 6A and D). In the renal proximal tubule of the rat, it is known that dopamine can inhibit NKA activity by activating DRD1 to regulate the renal sodium excretion^44^,^45^. Thus, we sought to explore the functions of *SaDRD1* in the regulation of NKA activity in renal primary cells of the spotted scat by using RNAi to reduce *SaDRD1* expression. si-*SaDRD1* reduced the expression and protein levels of SaDRD1. This treatment also affected expression of the protein subunit NKA α1 and the activity of NKA, which was significantly higher in treated cells than in control group (Fig. 5A and B). NKA α1 is one of catalytic subtypes that cleaves high-energy phosphate bonds and exchanges intracellular Na^+^ for extracellular K^+^ions, and is predominantly expressed in the kidney^46^. Our results showed that the inhibition of *SaDRD1* expression can significantly increase NKA activity through upregulating the protein expression of NKA α1. Under isosmotic condition, exogenous dopamine (both 1 μM and 10 μM) stimulates the expression of *SaDRD1* in renal primary cells at 3 h after exposure. Higher expression of *SaDRD1* was observed in the group induced with 1 μM dopamine (18.9-fold) than with 10 μM dopamine (6.7-fold) (Fig. 6B). There are some evidences suggesting that dopamine can increase dopamine receptor expression in levodopa-treated mice^47^. In addition, recruitment of DRD1 protein to the plasma membrane is also regulated by dopamine^48^. Thus, our results imply that dopamine can promote the high expression of *SaDRD1* in the short-term, and the SaDRD1 density is altered in plasma membrane through the recruitment from cytoplasm to the plasma membrane in a dose- and time-dependent manner.

The effects of exogenous dopamine (1 μM) on NKA activity in renal primary cells were explored. The results show that dopamine enhances the NKA response early when renal cells are faced with hyposaline stress by activating dopaminergic system through *SaDRD1* (Fig. 6C and D). The upregulation of *SaDRD1* mRNA occurred in a dose-dependent manner, with the higher dose of dopamine (10 μM) resulting in the blunted dopaminergic system activity, in accordance with a previous study in the rat^49^. Thus, we conclude that the activation of SaDRD1 by dopamine participates in FW acclimation by igniting dopaminergic system to inhibit the activity of NKA, leading to maintenance of osmotic homeostasis in the kidney of *S. argus*.

**Figure 7:**
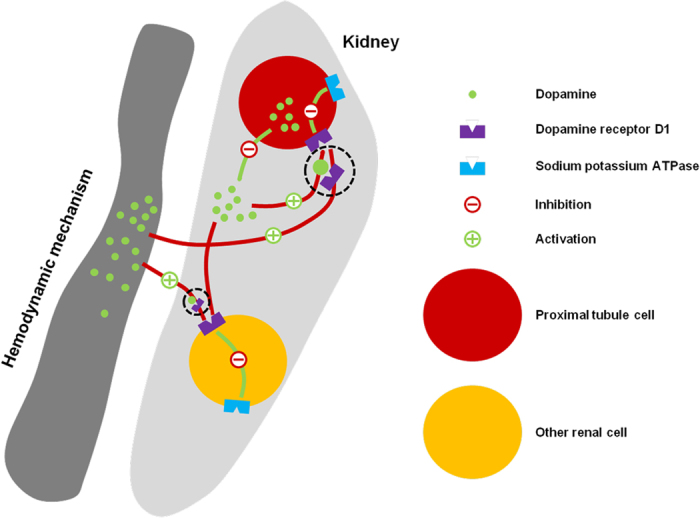
Our model of dopamine-mediated regulation on Na^+^/K^+^-ATPase (NKA) activity in the kidney of *S. argus*. During the acclimation to abrupt FW shock in *S. argus*, intrarenal dopamine was able to enhance early the response of NKA to hyposaline stress through SaDRD1.

During the acclimation to FW shock in *S. argus*, intrarenal dopamine plays an important role in the regulation of osmotic balance by activating renal SaDRD1, while SaDRD5 does not appear to be involved. SaDRD1 is expressed in the proximal tubule cells, and subcellularly localized to the cell membrane of cultured renal cells. During hypoosmotic shock of cultured renal cells, exogenous dopamine enhanced the early response of NKA to hyposaline stress through *SaDRD1*. Based on our results, we hypothesize that the dopaminergic system responds to hypoosmotic shock *via* DRD1 activation, which leads to inhibition of NKA activity and maintenance of osmotic balance in the fish exposed to acute hypoosmotic shock (Fig. 7). Further mechanistic studies are necessary to explore these intriguing hypotheses in detail.

## Methods

### Collection, maintenance, and treatment of fish and collection of tissue samples

*Scatophagus argus* (32.6 ± 7.4 g) were collected from Zhuhai (N22°09′16.82″, E113°21′41.50″), Guangdong Province, China and reared for 3 weeks in 25‰ seawater (SW, approximately 620 mOsmol/L) at 27 ± 2 °C. Fish for hyposaline challenge were divided into a treatment group (n = 105) and a control group (n = 105). Treated individuals were transferred from 25‰ SW to fresh water (FW, 0 mOsmol/L). Control individuals were transferred to another SW tank (identical salinity). Fifteen individuals were removed from each group at each experimental time point and anaesthetized^7^. Each set of 15 individuals was divided into 3 groups of 5 individuals, each group comprising 1 biological replicate. Samples were collected at 1 hour post-transfer (hpt), 3 hpt, 6 hpt, 12 hpt, 24 hpt, 2 days post-transfer (dpt), and 7 dpt, and inlcuded brain, kidney, gill, eye, liver, spleen, notochord, skin, intestines, heart, muscle, pituitary, ovary, spermary and blood. Tissue was frozen in liquid nitrogen and stored at −80 °C. Serum was separated from blood cells by centrifugation (4200 × g for 10 min) and assayed for dopamine concentration. Animal welfare and experimental procedures were in accordance with the Guide for the Care and Use of Laboratory Animals (Ministry of Science and Technology of China, 2006) and were approved by the animal ethics committee of Shanghai Ocean University.

### Preparation and culture of primary kidney cells

Kidney tissue was collected from healthy SW-acclimated fish weighing ~40 g and used for primary cell culture as described in Zhou *et al*.^36^. The cultures of primary cells were maintained at 28 °C. Approximately 80% of the primary cells adhered to the culture plate.

For hypoosmotic stress experiments, primary cells were subjected to hypoosmotic medium (100 mOsmol/L), while control cultures were exposed to fresh isosmotic medium (300 mOsmol/L) (survival rate >95%). Culture medium and cells were collected at 0.5 h and 3 h post-challenge, respectively, and used for hormone assays, RNA extraction and NKA activity measurement.

Additional cultures of primary cells in isosmotic medium were maintained at a density of approximately 2 × 10^4^/cm^2^ at 28 °C for transfection and immunohistochemistry staining.

### Dopamine detection

Dopamine in serum and culture medium was measured by indirect enzyme linked immunosorbent assay (ELISA) as described by Wu *et al*.^50^ using the Fish Dopamine ELISA Kit (CUSABIO, China) according to the supplier’s instructions. Negative controls (PBS-only and blank) were included in the assay. After the reaction, the optical density of each well was detected at 450 nm within 10 minutes using Synergy H4 Hybrid Multi-Mode Microplate Reader (BioTek, USA). Dopamine concentration of each sample was calculated according to a standard curve^7^.

### Total RNA isolation and cDNA synthesis

Total RNA was isolated using Trizol Reagent (Invitrogen, USA) following the manufacturer’s protocol. RNA was treated with DNase I (Invitrogen, USA) and quantified *via* Nanodrop-2000 spectrophotometer (Thermo Scientific, USA). RNA integrity was verified by gel electrophoresis. Only samples with A260/A280 ratios 1.8 to 2.0 were used. cDNA was synthesized by reverse transcription of 1 μg total RNA with oligo(dT) primers using the PrimeScript RT reagent kit with gDNA Eraser (Takara, Japan) and stored at −20 °C.

### Cloning of full-length *SaDRD1*

To obtain the partial sequences of *SaDRD1*, primers (Table 1) were designed using Primer Premier v5.0 (Premier, Canada) based on homologous sequences obtained from the National Center for Biotechnical Information (NCBI, http://www.ncbi.nlm.nih.gov/). *SaDRD1* was amplified from *S. argus* brain cDNA. PCR products were purified by gel extraction (Axygen, USA) and cloned into the pMD18-T vector (Takara, Japan) for sequencing.

To obtain the full-length cDNA sequence of *SaDRD1*, rapid amplification of cDNA ends (RACE) was performed (SMART RACE amplification Kit, Clontech, USA). Primers are listed in Table 1. The amplified PCR product was sequenced using an ABI PRISM 377 DNA Sequencer (Applied Biosystems, USA).

### Sequence analysis

The nucleotide sequence of *SaDRD1* was aligned with Clustal X 1.81 and analyzed using ORF Finder (http://www.ncbi.nlm.nih.gov/gorf/gorf.html) to predict the amino acid sequence and open reading frames (ORFs). Transmembrane domains were predicted using TMHMM v. 2.0 (http://www.cbs.dtu.dk/services/TMHMM-2.0/). A phylogenetic tree was constructed using MEGA 6.0 software with neighbor-joining method and 1000 boot-strap replicates^51^.

### Tissue distribution of *SaDRD1* mRNA

Semi-quantitative reverse transcriptase PCR (SqRT-PCR) was conducted to assess tissue expression patterns of *SaDRD1* in *S. argus*. Primers are listed in Table 2. The annealing temperature and PCR conditions were: 60 °C, 28 cycles for *β-actin*; 55 °C, 28 cycles for *SaDRD1*. The PCR products were visualized by 2% gel electrophoresis and expression was quantified using densitometric analysis (Image Lab, Bio-Rad, USA). *SaDRD1* expression was normalized against *β-actin*.

### Determination of SaDRD1 antibody specificity

Custom polyclonal antibodies were raised against SaDRD1 using a recombinant peptide (amino acids 336–463 of predicted SaDRD1 protein: NADFRKAFSILLGCHRLCPGSNAIEIVSINNNMGAPTSNPNCQYQPKSHIPKEGNHSSNYVIPHSILCQEEELQKKDACGGEIDVGMVNNALEKLSPAISGNLDSDTEVTLEKINPITQNGQHKAMSC) (YouKe, China).

Kidneys of *S. argus* and rat were homogenized on ice in normal saline (0.86% NaCl) and lysates were centrifuged at 12000 rpm 4 °C for 10 min. Supernatant was collected and protein concentration determined using a BCA Protein Assay Kit (Merck, Germany). About 75 μg total protein of *S. argus* or rat kidney was separated on 12% SDS-polyacrylamide gels and transferred to PVDF membranes. Following blocking in 5% skim milk in PBS (Bio-Rad, USA), membranes were incubated with rabbit anti-SaDRD1 (1:2000 dilution) in PBS overnight at 4 °C. Membranes were washed and incubated with horseradish peroxidase (HRP)-conjugated anti-rabbit antibody (Abcam, UK) (1:5000 dilution) for 2 h at room temperature. The signals were detected by chemiluminscence using Universal HoodII (Bio-Rad, USA).

### Immunohistochemistry

Kidney tissue samples collected from *S. argus* were fixed for 16 h in 4% paraformaldehyde at 4 °C, dehydrated using a graded series of sucrose solutions, embedded in Tissue-Tek O.C.T (Sakura, Japan), and cut into 20-μm sections at −20 °C. Sections were mounted on poly-l-lysine-coated slides (ShiTai, China), incubated in 5% skim milk for 1 h at 37 °C, incubated with primary antibody (1:400 dilution) in PBS containing 0.5% Triton X-100 (PBST) overnight at 4 °C, then incubated with FITC-conjugated goat anti-rabbit secondary antibody (Abcam, UK) (1:1000 dilution) in PBST for 3–4 h at the room temperature. Nuclei were stained with DAPI (300 nM, Sigma-Aldrich, USA) in PBS for 5 min.

Cultured primary cells were fixed for 15 min in 4% paraformaldehyde, incubated in 5% skim milk for 0.5 h at 37 °C, incubated with primary antibody (1:400 dilution) in PBS containing 0.5% Triton X-100 (PBST) for 3 h at 4 °C, then with FITC-conjugated goat anti-rabbit secondary antibody (1:1000 dilution) in PBST for 1.5 h at room temperature. Nuclei were stained with DAPI (as above) for 3 min.

Samples were visualized with a Zeiss LSM510 confocal laser scanning microscope (Zeiss, Germany). Negative controls were incubated with normal rabbit serum.

### Small interfering RNA (siRNA) and cell transfection

Small interfering RNA (siRNA) was employed to knock down expression of *SaDRD1* in spotted scat renal primary cells. Silencer select siRNA oligonucleotides 5′-GGUGCAUUCUGCAACGUAUtt-3′ (designated si-*SaDRD1*) against *SaDRD1* were designed and synthesized by Genepharma (Genepharma, China). A non-specific silencer select siRNA oligonucleotides (5′-CUCCGAACGUGUCACGUtt-3′; designated si-N.C.) served as negative controls. Cells were treated with siRNA in 6-well plates according to manufacturer’s instructions using Lipofectamine 3000 (Invitrogen, USA). Expression levels of the protein SaDRD1 and NKA activities were evaluated at 40 h post-transfection.

### Dopamine exposure of primary cells

Primary cells cultured in isosmotic medium (approximately 300 mOsmol/L) were shocked by hypotonic medium (approximately 100 mOsmol/L) containing 1 μM and 10 μM dopamine (H8502, Sigma-Aldrich, USA). Additional primary cells in original medium containing 1 μM and 10 μM dopamine were regarded as control groups. At 0.5 and 3 h post-challenge with dopamine, cells were washed with isosmotic/hypotonic medium and collected for RNA extraction and NKA activity measurement.

### Measurement of *SaDRD1* expression and NKA activity

*SaDRD1* mRNA expression was quantified by RT-qPCR. Reactions were performed in triplicate for each sample using SYBR Premix Ex Taq II (Takara, Japan). RT-qPCR primer sequences are listed in Table 2. All data was analyzed by the 2^−ΔΔCt^ method as described in Mu *et al*.^7^.

Immunostaining for SaDRD1 and NKA α1 of primary cells treated with siRNA was performed as above. Total protein was isolated and quantified at 40 h after *SaDRD1* siRNA treatment. Subsequently, NKA activity was measured following the protocol supplied by the manufacturer of the ATPase Kit (JianCheng, China). Activity was expressed as μmolPi/mgprot/h.

### Statistical analysis

The statistical significance of differences between groups was determined using the One-way ANOVA, followed by student’s *t*-test with SigmaStat software (Systat Software, USA). * means significant (*p* < 0.05), and *** means significant (*p* < 0.001).

## Additional Information

**How to cite this article**: Su, M. *et al*. Dopamine regulates renal osmoregulation during hyposaline stress *via* DRD1 in the spotted scat (*Scatophagus argus*). *Sci. Rep*. **6**, 37535; doi: 10.1038/srep37535 (2016).

**Publisher’s note:** Springer Nature remains neutral with regard to jurisdictional claims in published maps and institutional affiliations.

## Supplementary Material

Supplementary Information

## Figures and Tables

**Table 1:** Primers used to clone the gene *SaDRD1*.

Primer name	Primer sequences	Purpose
*SaDRD1*-partial-F	5′-GGTRGCCTTTGACATCATGT-3′	Partial *SaDRD1* cloning
*SaDRD1*-partial-R	5′-TAGATVCKGGTGTARGTGAC-3′	Partial *SaDRD1* cloning
*SaDRD1*-3′RACE-GSP1	5′-GAGAGTGCCAAAAACCGCCA-3′	3′ RACE for *SaDRD1*
*SaDRD1*-3′RACE-GSP2	5′-TAGATGATGGGGTTGAGCGAGG-3′	3′ RACE for *SaDRD1*
*SaDRD1*-5′RACE-GSP1	5′-CTGCTGGTTGCCCTTCTTCATCCTT-3′	5′ RACE for *SaDRD1*
*SaDRD1*-5′RACE-GSP2	5′-TTCAGGCTGGAATCGCAGTTGTC-3′	5′ RACE for *SaDRD1*

**Table 2:** Primers used for quantitative and semi-quantitative RT-PCR.

Primer name	Primer sequences	Product size (bp)
*SaDRD1*-semi-F	5′-CCATCTCAAGCCCATTTCGC-3′	542
*SaDRD1*-semi-R	5′-GGCACCTTCAGGCAAGTTCG-3′	
*SaDRD1*-qRT-F	5′-AGCCCATTTCGCTATGAACGCA-3′	192
*SaDRD1*-qRT-R	5′-TCAGGCTGGAATCGCAGTTGTC-3′	
*SaDRD5*-qRT-F	5′-CATGGTGGTGTCGTGGTGAT-3′	142
*SaDRD5*-qRT-R	5′-CCCGGTCATTTACGCCTTCA-3′	
*NKA α1*-qRT-F	5′-AGCTGAAAGACATGACCGCA-3′	183
*NKA α1*-qRT-R	5′-TGTCAGCCTTCTTCAGAGCG-3′	
*β-actin*-F	5′-TAGTCTGTGAGGTCACGG-3′	151
*β-actin*-R	5′-CTGTGCTGTCCCTGTATG-3′	

B = C/G/T, H = A/T/C, K = G/T, M = A/C, R = A/G, S = C/G, V = A/C/G, W = A/T, Y = C/T.
