# Supplementary material for: Dopamine regulates renal osmoregulation during hyposaline stress via DRD1 in the spotted scat (Scatophagus argus)
Source: Sci Rep. 2016 Nov 18;6:37535. doi: 10.1038/srep37535 (PMC5114590; doi:10.1038/srep37535)
Supplement: Supplementary Information [file srep37535-s1.pdf]

Supplementary Information

Dopamine regulates renal osmoregulation during hyposaline stress *via* DRD1 in the spotted scat (*Scatophagus argus*)

Maoliang Su[1], Xingjiang Mu[1], Lang Gui[1], Peipei Zhang[1], Jianan Zhou[1], Jie Ma[1] & Junbin Zhang[1]

[1]Key Laboratory of Exploration and Utilization of Aquatic Genetic Resources, Ministry of Education, College of Fisheries and Life Science, Shanghai Ocean University, Shanghai 201306, China.

	Control group		Treatment group	
	Gene name	Ct value ± S.E.M.	Gene name	Ct value ± S.E.M.
1hpt	*β-actin*	16.23±0.05	*β-actin*	16.12±0.08
	DRD1	28.50±0.02	*DRD1*	28.04±0.03
3hpt	*β-actin*	16.19±0.10	*β-actin*	16.09±0.03
	DRD1	29.36±0.05	*DRD1*	28.23±0.25
6hpt	*β-actin*	16.51±0.08	*β-actin*	16.71±0.02
	DRD1	28.58±0.18	*DRD1*	28.59±0.03
12hpt	*β-actin*	16.06±0.05	*β-actin*	16.44±0.15
	DRD1	29.41±0.03	*DRD1*	28.79±0.23
24hpt	*β-actin*	16.09±0.08	*β-actin*	16.09±0.09
	DRD1	28.67±0.08	*DRD1*	28.60±0.24
2dpt	*β-actin*	16.97±0.08	*β-actin*	16.95±0.07
	DRD1	30.28±0.04	*DRD1*	29.64±0.20
7dpt	*β-actin*	16.51±0.06	*β-actin*	16.63±0.04
	DRD1	29.54±0.07	*DRD1*	29.79±0.21

Table S1. Ct values of *SaDRD1* gene expression levels in the analysis of RT-qPCR data.

	Control group		Treatment group	
	Gene name	Ct value ± S.E.M.	Gene name	Ct value ± S.E.M.
1hpt	*β-actin*	16.92±0.15	*β-actin*	16.52±0.17
	DRD5	30.51±0.22	*DRD5*	30.40±0.25
3hpt	*β-actin*	16.96±0.14	*β-actin*	16.57±0.13
	DRD5	29.51±0.16	*DRD5*	28.39±0.21
6hpt	*β-actin*	16.89±0.23	*β-actin*	16.97±0.04
	DRD5	29.84±0.24	*DRD5*	ND
12hpt	*β-actin*	16.98±0.05	*β-actin*	17.13±0.06
	DRD5	30.36±0.21	*DRD5*	29.75±0.12
24hpt	*β-actin*	16.88±0.11	*β-actin*	16.98±0.16
	DRD5	31.74±0.09	*DRD5*	32.08±0.28
2dpt	*β-actin*	16.79±0.06	*β-actin*	16.67±0.11
	DRD5	31.10±0.12	*DRD5*	ND
7dpt	*β-actin*	16.32±0.08	*β-actin*	16.22±0.10
	DRD5	31.32±0.05	*DRD5*	ND

Table S2. Ct values of *SaDRD5* gene expression levels in the analysis of RT-qPCR data. "ND" represents "not bedetected".

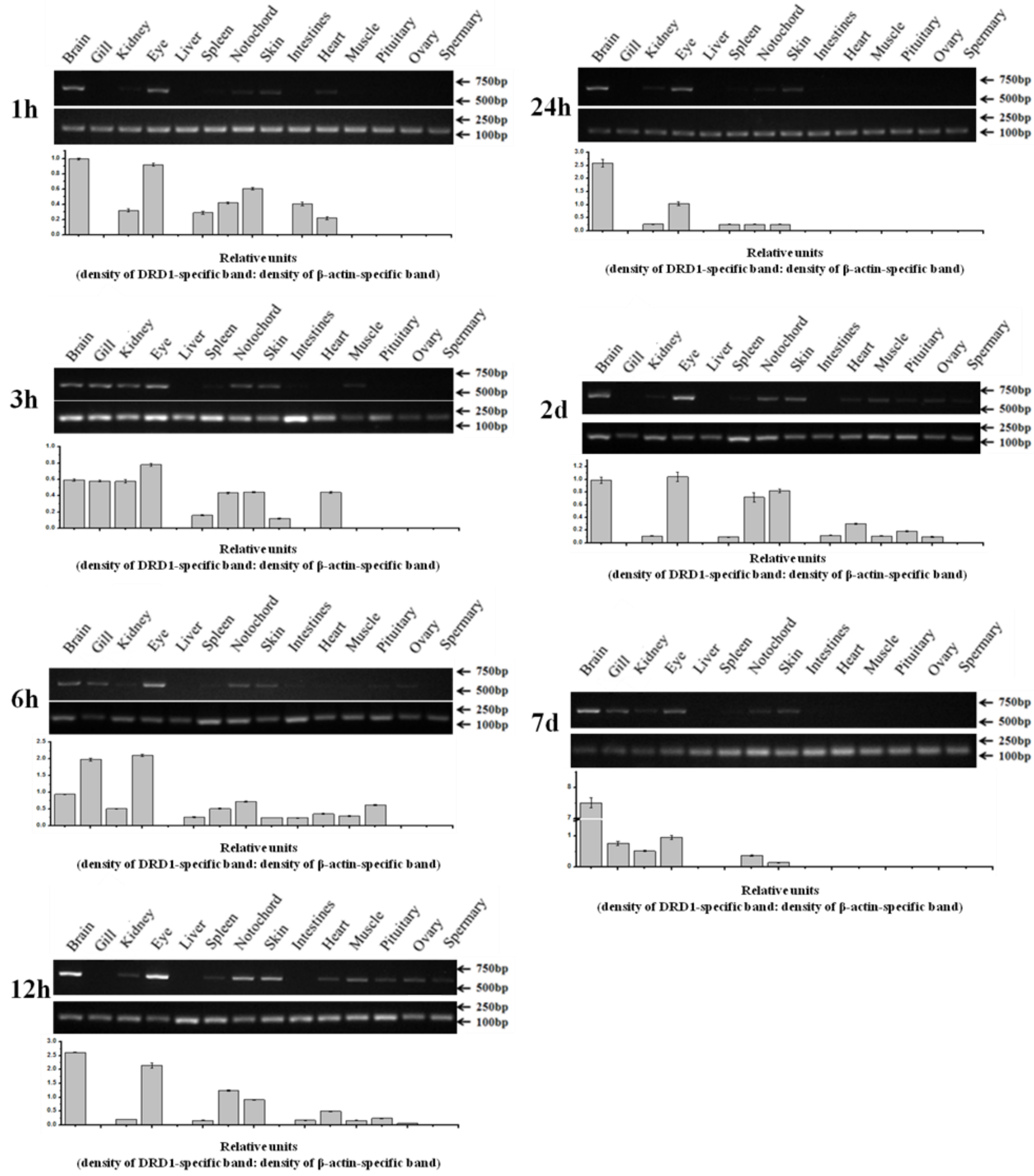

Figure S1. Tissue distributions of *SaDRD1* gene after FW-shock at different time points. The results are presented as relative units (density of *DRD1*-specific band versus density of *β-actin*-specific band).

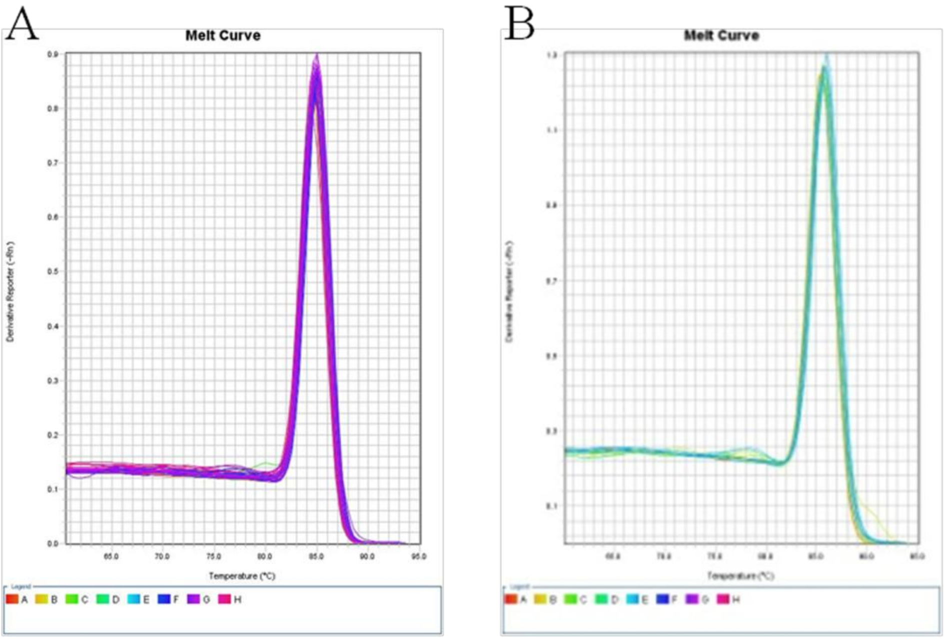

Figure S2. The melt curve of *SaDRD1* (A) and *SaDRD5* (B) in RT-qPCR.
